# Editorial: Patterns, functions, and processes of alpine grassland ecosystems under global change

**DOI:** 10.3389/fpls.2022.1048031

**Published:** 2022-10-12

**Authors:** Jian Sun, Yingxin Wang, Shiliang Liu, Junran Li, Huakun Zhou, Gaolin Wu, Nigussie Haregeweyn

**Affiliations:** ^1^ State Key Laboratory of Earth System Resources and Environment of Tibetan Plateau, Institute of Tibetan Plateau Research, Chinese Academy of Sciences, Beijing, China; ^2^ State Key Laboratory of Water Environment Simulation, School of Environment, Beijing Normal University, Beijing, China; ^3^ Department of Geography, The University of Hong Kong, Hong Kong, Hong Kong SAR, China; ^4^ Key Laboratory of Restoration Ecology for Cold Regions in Qinghai, Northwest Institute of Plateau Biology, Chinese Academy of Sciences, Xining, China; ^5^ State Key Laboratory of Soil Erosion and Dryland Farming on the Loess Plateau, Institute of Soil and Water Conservation, Northwest A&F University, Yangling, China; ^6^ International Platform for Dryland Research and Education, Arid Land Research Center, Tottori University, Tottori, Japan

**Keywords:** patterns, functions, processes, alpine grassland, global change

Alpine grassland on the Tibetan Plateau (TP) accounts for about 62% of the total area of the TP (Wang et al., 2022). These grassland ecosystems provide important ecological services and functions in pastoral production, biodiversity conservation, carbon storage, water resource regulation, cultural inheritance, tourism, and recreation etc. (Miehe et al., 2019; Sun et al., 2020; Wang et al., 2022). In the past serval decades, the alpine grassland on the TP has experienced rapid climate change and intensified human activities (He et al., 2021), the regulatory mechanisms and drivers are remained controversial.

Climate changes have been evident across the TP since the beginning of the 1970s (Yang et al., 2014). For instance, the mean annual air temperature has increased by about 0.4°C, which is twice that of the global average, however, a decrease in precipitation has been found in many other regions (Mountain Research Initiative EDW Working Group, 2015). Also, the degradation of permafrost has been accelerated as manifested by ground surface subsidence and thaw settlement of permafrost soils (Mu et al., 2020), and the total number of lakes increased from 868 in 1990 to 1207 in 2015, leading to the increase of total water surface area of the lakes at a rate of 383.5 km2 yr-1 (Sun et al., 2018). With the steady increase of human population and domestic livestock number (Wei et al., 2020), overgrazing has become a serious challenge with overstocking rates of 27–89% in the alpine grassland (Wang et al., 2022). Currently, there are approximately 20 million yak and 50 million Tibetan sheep grazing in the alpine grassland, making it one of the regions with high grazing pressure worldwide (Wang et al., 2020). The interactions of soil, plant, animal, and grazing management affect the production and stability of grassland ecosystems, and also provide the driving force for the functions and services of the alpine grassland on the TP (Hodgkinson and Wang, 2020; Sun et al., 2022). For example, grazing exclusion has been widely adopted by the local and central government, predominantly through the use of fences, to restore the degraded alpine grasslands since 2003 (Sun et al., 2021). The total area of fences had increased up to 3.32 million hectares on the northern Tibetan Plateau (Sun et al., 2020).

In the past 20 years, numerous efforts have been done to explore the patterns, functions, and processes of alpine grassland ecosystems, including transect surveys, field observations, model simulation, comprehensive meta-analyses, and literature reviews. These studies have following characteristics: (i) with the improvement of testing methods, a diversity of technologies have been applied at the technical level such as remote sensing, model simulation, large-scale transect sampling survey, aerial filming, and long-term observation. Scales of these studies range from regional, landscape, ecosystem, and community to micro-molecular and genomic levels; (ii) the observation indexes have evolved from basic to comprehensive, which includes moisture, soil, biology, watershed, and climate; (iii) various factors such as the environmental, natural, human activities, and the impact of the whole habitat on alpine ecosystems were taken into account, and (iv) studies have evolved from trophic levels, food webs, and independent processes of ground and underground to the integration of above and below ground processes of the whole ecosystem. Despite remarkable knowledge gains, there are still some areas that require in-depth research, such as the driving mechanism of the impact of global change alpine ecosystems with new technologies and theories.

To advance our understandings of the patterns, functions, processes, and mechanisms of alpine grassland ecosystems responding to changing environments (e.g., dynamics of temperature and precipitation, nitrogen deposition and extreme weather events, grazing, fencing and ecological projects, etc.)., we organized a Special Issue (SI) entitled “Patterns, Functions, and Processes of Alpine Grassland Ecosystems under Global Change”. This SI comprises 49 articles, sharing new ideas, techniques, and findings about this subject from field experiments, large-scale transect surveys, alongside remote sensing (Wang et al., 2022a; Zhang et al., 2022; Zou et al., 2022). These studies shed light on the future development of adaptive management practices for alpine grassland ecosystems under future environmental change.

The spatial-temporal patterns and drivers of alpine grassland ecosystem under the changing environment are the top priority for the grassland conservation. Due to the scale of the TP, remote sensing is critical for studies on the spatial and temporal heterogeneity of the grassland. Four papers in this SI discuss the spatial-temporal patterns and drivers of alpine grassland ecosystem under the changing environment based on patch, landscape or pixel based remote sensing observations. Zou et al. (2022) used neural networks and spatiotemporal indices fusion method to analyze the dynamic disturbance analysis of grasslands on the TP. Fan et al. (2021) carried out a 5,000 km belt transect investigation to distinguish stoichiometric homeostasis of soil microbial biomass in alpine grassland ecosystems. Huang et al. (2022) estimated the net ecosystem exchange (NEE) using the improved Biome-BGCMuSo model to characterize the spatiotemporal dynamics of the carbon budget. Shi et al. (2022) evaluated the effects of climate change on the distribution of Meconopsis punicea and ecosystem services supply.

Seven papers in this SI assess the variations of alpine ecosystem functions, and the response and feedback of ecological processes using long-term simulated experiments and large-scale transect surveys. Some of the studies used the entire TP as the study area and evaluated the complex biodiversity-ecosystem function relationships (Zhang et al., 2022; Qi et al., 2022), the effects of soil physico-chemical properties on plant species diversity (Han et al., 2022), the mechanism of plant communities on different EMF patterns (Wang et al., 2021), and on the canopy water use efficiency (Li et al., 2022b). Other studies investigated the impacts of human activities on ecosystems, especially alpine grasslands and meadows, with regard to overgrazing, rodent destroy, and grassland degradation. For example, Wang et al. (2022a)> indicated that climate change or the phenological change has stronger effects on ANPP. Li et al. (2021a) demonstrated that small semi-fossorial herbivores have an important impact on the spatial heterogeneity of vegetation.

Climate change and human activities dominate the recent changes in grassland patterns and processes on the TP, thus affecting the ecosystem functions and services. Many of the articles in this SI reveal the driving mechanism of the impact of climate change and human activities (Dai et al., 2022; Hua et al. (2022); Ma et al., 2022; Zuo et al., 2022) on alpine ecosystems with field observations and manipulative experiments combined with new experimental technologies (Chen et al., 2022; Qin et al., 2022; Zou et al., 2022). Experimental warming enhanced the pattern of the plant allometric growth and promoted migration of the soil stoichiometric characteristics to deep layers in Beiluhe (Xu et al., 2022). However, in Haibei, Yang et al. (2022) indicated that the warming treatment has not affected the changes in soil organic carbon. In addition, extreme drought can affect Re and Rs by altering plant and soil extracellular enzyme activities in alpine wetland (Yan et al., 2021). In alpine meadow, N and P addition significantly affect t plant communities (Ma et al., 2021), soil microbial community and enzyme activities (Zi et al., 2022), and microbial functional genes (Xiao et al., 2022). In alpine steppe, N and P application led to an increase in aboveground biomass (Li et al., 2021b), and increased intensity of bacteria and fungi (Li et al., 2021c), also affected the plant functional traits (Li et al., 2022d). Meanwhile, grazing increased herbage respiration in summer, presumably through stimulation of gross photosynthesis (Yuan et al., 2022), and changed plant community composition and reduced stochasticity of soil microbial community assembly of alpine grasslands (Li et al., 2022a). Restoration of degraded grassland significantly improves shoot and root biomass (Zhang et al., 2022), plant interspecific associations (Wu et al., 2022), soil enzymatic activities (Wang et al., 2022b), and water storage (Guo et al., 2021).

Seven papers in this SI explored the mechanisms of alpine ecosystem dynamics using new analytical methods, e.g., artificial intelligence learning, network analysis (Zhou et al., 2022), Bayes analysis and other data mining methods (Zhou and Peng (2021). Dong et al. (2021) used phylogenetic correlation and symbiotic network to explain the interdependence between plants and arbuscular mycorrhizal fungi in alpine meadow. Also, the degradation of alpine meadows mediated by climatic factors and effects of different grazing regimes on the plant diversity, plant community traits and ecological functions were analyzed by using meta-analysis (Li et al., 2021d; Yu et al., 2022; Zhan et al., 2022). Wang et al. (2022c) highlighted that ecosystem coupling and ecosystem multifunctionality may be used to evaluate the plant succession induced by grazing.

It is well known that human activities and their feedbacks affect regional sustainable development, but relevant policy measures have rarely be evaluated at the household level. Hua et al. (2022) explored the views on grassland restoration programs on the Qinghai-Tibet Plateau. Also, feedback and trigger of household decision-making to ecological protection policies in Sanjiangyuan National Park were studied (Su et al., 2022).

Although some findings on the ecological system of TP may still be somewhat preliminary, all studies included in this SI have undoubtedly promoted our understanding of the patterns, functions, and processes of alpine grassland ecosystems in the context of global change. Some important questions remain unanswered, though. Further explorations need to focus on the following two aspects: (i) more long-term localized observations and manipulative experiments should be employed to reveal long-term change of coupling of plant-soil-livestock-human system and its driving mechanisms. Two TP-wide programs, namely, the Alpine Fence Observation Net (AFON) and the Alpine Climate Change Observation Net (ACON) were established in 2020 and 2022, respectively (**Figure 1**). The AFON contains 53 fenced observation sites in alpine meadow, alpine steppe and alpine desert steppe across the TP. Likewise, the ACON contains 23 study sites with the unified climate change experiment, such as warming (Open-top-chamber), N addition, removing the light and removing the plant functional group. (ii) special attentions must be drawn upon the ecological network of multiple trophic levels of alpine grassland and their responses to global change. There is a lack of empirical evidence in this respect. Encouragingly, with the advancement of science and technology, such as network analysis tools, access to internet in remote areas, molecular technologies (e.g., meta-genomics, and - metabolomics), the study of aboveground-belowground interactions of ecosystems is expected to make great progress in the near future.

**Figure 1:**
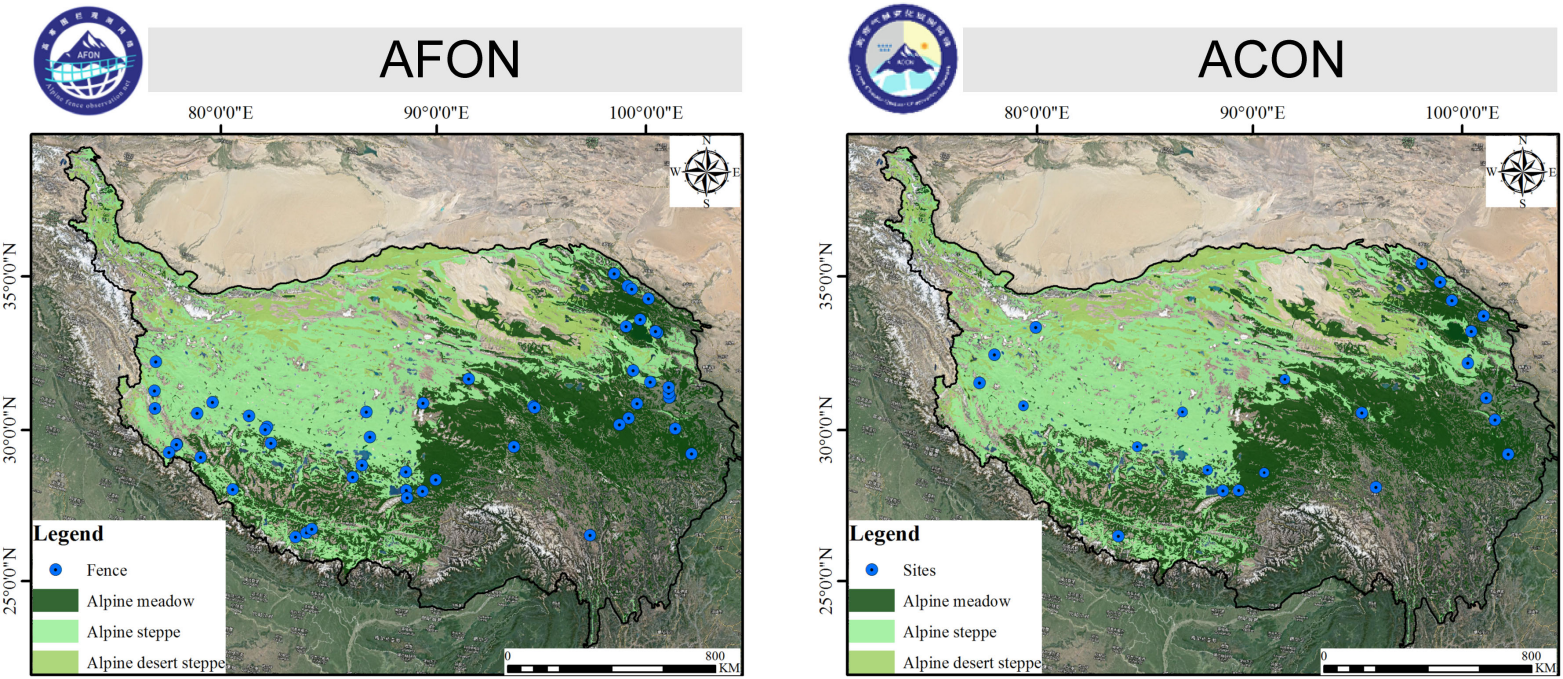
Locations of the Alpine Fence Observation Net (AFON) and the Alpine Climate Change Observation Net (ACON) on the TP. Both AFON and ACON are constructed by Prof. Sun’ team at the Institute of Tibetan Plateau Research, Chinese Academy of Sciences.

## Author contributions

JS, YW, SL, and JL drafted the story line of the editorial. SL, HZ, GW and NH refined the story line and contributed references and insights to the impact of the papers included in this editorial. All authors contributed to the article and approved the submitted version.

## Funding

This work was supported by the National Natural Science Foundation of China (No. 41871040), the National Natural Science Foundation of China Joint Fund Project (U21A20186), the Joint Research Project of Three-River-Resource National Park Funded by the Chinese Academy of Sciences and Qinghai Provincial People’s Government (LHZX-2020-08), Qinghai Natural Science Fund Innovation Team Project (2021-ZJ-902).

## Acknowledgments

We thank all the authors who submitted their work to this Research Topics, the professional editorial staff at Frontiers in Plant Science for their support in creating this Research Topic, and the invaluable help of reviewers in manuscript evaluation.

## Conflict of interest

The authors declare that the research was conducted in the absence of any commercial or financial relationships that could be construed as a potential conflict of interest.

## Publisher’s note

All claims expressed in this article are solely those of the authors and do not necessarily represent those of their affiliated organizations, or those of the publisher, the editors and the reviewers. Any product that may be evaluated in this article, or claim that may be made by its manufacturer, is not guaranteed or endorsed by the publisher.
